# Using a Transdisciplinary Approach in Learning Communities for Designing Wearable Stress Management for Vulnerable Populations: Development and Usability Study

**DOI:** 10.2196/75836

**Published:** 2026-01-09

**Authors:** Manon W H Peeters, Ittay Mannheim, Paula Elisabeth van Westrienen, Leoni van Dijk, Annalisa Elia, Karolina Valterova, Evelien van de Garde-Perik, Petra Heck, Noortje Lavrijssen, Gerard Schouten, Eveline J M Wouters

**Affiliations:** 1 School for Allied Health Professions, Fontys University of Applied Sciences, Rachelsmolen 1, Eindhoven, 5612MA, The Netherlands; 2 Department of Communication Studies, Ben-Gurion University of the Negev, Be'er Sheva, Israel; 3 School for Information and Communication Technology, Fontys University of Applied Sciences, Eindhoven, The Netherlands; 4 Department of Law and Digital Technology, Juridische Hogeschool Avans-Fontys, Den Bosch, The Netherlands; 5 School of Social and Behavioural Sciences, Tilburg University, Tilburg, The Netherlands

**Keywords:** transdisciplinary research, learning communities, wearable technology, stress monitoring, participatory design, digital health

## Abstract

**Background:**

Software solutions for wearable-based stress monitoring offer significant potential in health care, particularly for vulnerable populations such as individuals with dementia or persistent physical symptoms. Despite technological advances, designing user-centered, ethically grounded, and contextually relevant software remains challenging. Vulnerable populations often have specific cognitive, physical, and emotional needs that require customization, yet these are rarely prioritized in mainstream development. Our so-called Sensors2Care project addressed these challenges by co-developing stress-monitoring prototypes in collaboration with stakeholders from health care, law, and technology within a transdisciplinary setting.

**Objective:**

This article has two aims: first, to describe how the Sensors2Care project operationalized the transdisciplinary approach (TDA) within a learning community (LC) to guide the development of stress-monitoring software; second, to share stakeholder needs and design requirements for wearable technologies in complex health care contexts, derived from this process.

**Methods:**

The Sensors2Care project applied a TDA embedded in an LC. This approach combined participatory design research with mixed methods across 3 iterative components: requirements gathering, prototype development, and early-stage evaluation. Research activities included scoping reviews, semistructured interviews, focus groups, legal analyses, and field testing. In the LC, students and researchers from health professions, computer science, and law collaborated with patients, (in)formal caregivers, and industry partners in a transdisciplinary consortium. User stories served as both a methodological tool and design outcome, helping to capture stakeholder needs and align input from technical, health, and legal domains. Feedback was collected continuously and used to refine requirements and prototypes throughout the development process.

**Results:**

User stories revealed 7 key themes relevant to developing and using wearable-based stress monitoring, including strategic use, notifications, user input, data insight, data access and sharing, hardware design, and support. Stakeholders emphasized the need for customization, durability, and comfort, aligned with the cognitive and physical needs of the target populations. Prototype evaluations indicated the practical relevance of these features and revealed a need for training and insight into long-term usability. Beyond their role in capturing content-driven input, user stories also supported transdisciplinary collaboration by aligning legal, health, technical, and experiential perspectives. This was facilitated by the LC structure, which enabled sustained engagement between students, researchers, and societal stakeholders and illustrated the feasibility of implementing TDA in a university context.

**Conclusions:**

This project illustrates how TDA, when embedded in an LC, supports the co-development of ethically grounded, contextually relevant, and practically applicable stress-monitoring software for vulnerable populations. The iterative design process enabled early integration of legal, health, and technical considerations, while user stories supported structured collaboration across domains. Although the project resulted in concrete prototypes and clustered design requirements, further research is needed to assess long-term use and real-world implementation across health care contexts. Embedding TDA in LCs may strengthen future professionals’ ability to address complex health care challenges collaboratively.

## Introduction

### Overview

Technological advancements in wearable devices, data acquisition, and algorithmic processing (eg, machine learning) are transforming health care by enabling continuous and minimally invasive health monitoring [1]. Wearable devices are increasingly studied in health care, for instance, in smart health monitoring systems [2]. By being worn directly on the skin at various locations (eg, wrist, finger, chest, ankle), these devices facilitate continuous data collection, providing valuable insights into both physical and mental health parameters. Notably, they allow the tracking of stress, an essential factor in health management, through physiological markers such as heart activity and skin conductance [1]. The ability to monitor stress in daily practice in a minimally invasive way holds a particular promise for improving care and outcomes for individuals with complex health needs.

Despite this promise of wearable devices, their design and implementation often focus on younger, healthier populations, leaving vulnerable groups underserved [1,3]. Wearables for health care are typically developed for general lifestyle improvements [4], rather than addressing the specific needs of vulnerable populations, in health and care contexts. The complexity of designing systems that are both effective and feasible for these populations highlights the need for inclusive approaches. Designing and implementing wearables in diverse, real-life, care settings remains preliminary, complex, and requires cross-sectoral collaborations between academia, health care, industry, and users [5,6].

Recent reviews and studies provide further evidence of these challenges. Most work has concentrated on technical feasibility and algorithmic accuracy, with relatively little attention to usability, acceptability, and long-term adoption in health care contexts [2]. Although an increasing number of wearable devices are available, research on their validity, reliability, and usability, including user-friendliness and user acceptance, remains limited [7]. Evidence involving vulnerable populations shows both potential and barriers: Wearable biofeedback, for example, has shown promise for people with mild intellectual disabilities, but required substantial caregiver support and was difficult to integrate into daily routines [8]. Moreover, stress-tracking technologies often produce non-transparent scores that are difficult to interpret, increasing the risk of misinterpretation or misguided actions based on misleading feedback [9]. These challenges highlight the responsibility of designers to ensure that wearable feedback is meaningful and supportive.

To facilitate inclusive designs in health care, this study introduces the Sensors2Care project*,* which demonstrates how a transdisciplinary approach (TDA) can be applied within learning communities (LC) through 2 use cases of vulnerable populations.

### Sensors2Care Project

Designing wearables for stress monitoring involves making choices that need to balance technical possibilities with contextual requirements. Studying multiple use cases helps to distinguish context-specific from general requirements, strengthening the relevance of resulting design insights. Early identification and monitoring of stress are particularly beneficial for persons with various health conditions [2,10], highlighting the relevance of examining multiple contexts. The Sensors2care project, therefore, focused on 2 health care use cases: dementia and persistent physical symptoms (PPS).

Dementia is often associated with challenging behaviors [11] like anxiety and agitation, influenced by underlying stress [12,13]. These behaviors can negatively impact the quality of life of individuals and place a heavy burden on caregivers [14-16]. The other use case, PPS, includes distressing somatic complaints, such as pain and fatigue, that last several months or more, regardless of their cause. These symptoms occur in the context of somatic diseases, functional somatic disorders, mental disorders, and undiagnosed conditions and are a core problem in a wide range of medical disciplines [17]. PPS is characterized by a close interplay between symptoms and stress regulation: difficulties in perceiving bodily signals [18], regulating emotions [19,20], and maintaining autonomic balance [21] may exacerbate stress responses, which in turn can worsen symptoms [22]. These symptoms often limit daily functioning and create a substantial burden for patients and health care providers. In both use cases, PPS and dementia, stress management is already part of usual care [23,24], as timely stress identification can support tailored interventions, improve well-being, and reduce care burden.

Stress can be assessed using multiple methods, such as wearable devices that provide objective physiological data [2], caregiver observations, or self-reports like the Perceived Stress Scale [25]. The use of stress data depends on the context: individuals may use it for self-management and personal insights (quantified me), while (in)formal caregivers may monitor and support others (quantified you). Wearable devices enable real-time, remote stress monitoring, offering potential for timely interventions and quality of life improvements. However, prior research on wearables has mainly demonstrated technical feasibility, while challenges of usability, integration into daily routines, and long-term adoption remain insufficiently addressed [2,7]. Designing a wearable-based system that meets the practical and contextual needs of vulnerable populations and can be implemented successfully remains a complex challenge.

The Sensors2Care project explores how wearable-based stress-related measurements could be integrated into health care by developing prototypes that address social, technical, legal, and clinical requirements throughout the design process. The project combined TDA principles with LCs to address the complex challenges of designing stress-measurement wearables for vulnerable populations. Dementia care often relies on caregivers (quantified you) to interpret stress data, whereas PPS emphasizes the role of self-monitoring (quantified me) to empower individuals in managing their symptoms. Together, these contrasting yet complementary use cases provide a diverse testing ground for research and design activities informed by TDA. Considering both use cases in parallel allowed for a systematic analysis of requirements across different care contexts. This strengthened the robustness and practical relevance of the design insights.

### Transdisciplinary Approach

Designing and implementing wearable-based systems in real-life care settings involves challenges that extend beyond technology. The Non-adoption, Abandonment, Scale-up, Spread, Sustainability (NASSS) framework [26] highlights how implementation of innovative health care technologies frequently fails when challenges in key domains remain unaddressed, including the technology domain (eg, feasibility, interoperability), the value proposition and adopter system (eg, legal compliance, data governance), and the condition and context (eg, clinical relevance and fit with care practices). These domains represent common barriers to sustainable adoption in complex care settings and are therefore essential to consider in early stages of design and development.

Addressing these domains requires cross-sectoral collaboration, demanding approaches that integrate diverse types of knowledge and experience. TDA is particularly suitable for this task [27]. TDA extends beyond interdisciplinary research by integrating academic knowledge from diverse disciplines and incorporating non-academic perspectives, such as those of caregivers, health care providers, and technology companies [28]. By combining scientific, experiential, and local knowledge, TDA supports the co-creation of solutions that are relevant, robust, and practicable, thereby facilitating adoption and implementation in practice.

Despite the increasing number of studies on TDA, debate remains regarding its definition and methodological application. For an overview of diverse TDA definitions and shared characteristics, see [28]. A core characteristic of TDA is the integration of multidisciplinary and non-academic knowledge to address complex real-world problems, enabling context-sensitive and actionable insights grounded in scientific, professional, and lived experience. Another key characteristic of TDA is reflexivity. The continuous consideration of the broader research context and the alignment of project components and activities throughout the process. Such reflexive practice supports coherence within complex collaborations and the joint development of robust and societally relevant knowledge [27,28]. Through cross-sector collaboration, TDA offers a framework to design solutions tailored to the needs of vulnerable populations, such as individuals with dementia and PPS. Designs that are informed by such integrative processes are more likely to be accepted, adopted, and sustained in real-world care settings [26,28].

In the Sensors2Care project, TDA functioned as an overarching framework that was operationalized through participatory design approaches. Participatory methods, including human-centered design [29], generative design research, and the use of probes and prototypes [30], emphasize collaboration by actively involving end users to shape interventions that address their specific needs and circumstances [31]. Within TDA, participatory approaches provide concrete practices for co-creation, while TDA itself complements them by integrating knowledge across disciplinary and sectoral boundaries and fostering collaboration at multiple levels [27,28,32].

### Learning Communities

LCs play a key role in facilitating TDA by providing a collaborative framework for knowledge exchange and co-production. In this article, an LC is considered a structured, participatory environment where diverse stakeholders, including students, professionals, researchers, and experts by experience, engage as co-learners to share knowledge, address disciplinary barriers, and collaboratively develop innovative solutions. Cundill et al [33] describe how communities of practice facilitate TDA by enabling external partners to engage with core group activities, thereby addressing disciplinary power imbalances. Rather than being artificially created, such communities are nurtured through mutual engagement and collaboration. Within TDA, co-producing knowledge is viewed as a process where experts from different fields come together as co-learners to develop a “shared vocabulary“ that fosters shared understanding and vision [34]. Learning is thus framed as a social process, embedded in practices that are central to successful knowledge exchange [35]. As such, LCs form a foundation for developing innovative and inclusive solutions to complex health care challenges.

While LCs have been recognized as valuable tools for transdisciplinary collaboration, their practical organization remains challenging, particularly when involving students from different disciplines. Challenges include ensuring equitable participation, sustaining meaningful collaboration, and managing the complexities of co-creation across academic and non-academic boundaries [33,35]. In transdisciplinary research, enabling students from diverse disciplines, such as health care, computer sciences, and law, to collaborate with professionals and researchers, provides a structured way to integrate perspectives and develop shared solutions to complex challenges.

As highlighted by Lawrence et al [28], more empirical examples are needed to demonstrate how transdisciplinary collaboration can be organized and implemented in practice. The purpose of this article is twofold: first, to show how the Sensors2Care project used LCs as a setting for applying transdisciplinary research, to develop a stress-monitoring system using wearables for individuals with dementia and PPS; and second, to share insights into stakeholder needs and design considerations that emerged from this approach. By detailing the project’s co-exploration and conceptual design stages [29,30], we illustrate how LCs can bring together students, researchers, and professionals to foster knowledge exchange and collaboration in transdisciplinary research projects.

## Methods

### Organization of the Learning Community

In the 2-year Sensors2Care project, TDA was adopted within our LC. The project was explicitly designed to integrate academic and non-academic knowledge across scientific, professional, and experiential domains [27,28]. Disciplines relevant for both prototype development and implementation [26], including health, computer science, and law, were represented by researchers and students as future professionals. These domains were included based on known implementation challenges described in the NASSS framework [26], ensuring that technical, organizational, and contextual barriers were methodically addressed in the design process. This design was guided by the TDA principle that “rigor in applied science meets societal relevance” [28]. Additionally, the LC involved non-academic stakeholders, including (in)formal caregivers and patients from 4 health care institutions (2 specializing in PPS treatment and 2 nursing homes for individuals with dementia) and 6 small to medium enterprises (SMEs) in the IT sector. The LC functioned as a collaborative and reflexive environment in which participants considered the broader project context, aligned activities, and integrated diverse insights to co-produce knowledge. Together, these academic, professional, and experiential contributors represented diverse knowledge domains, operationalizing TDA’s principle of co-production across these domains.

**Figure 1.**
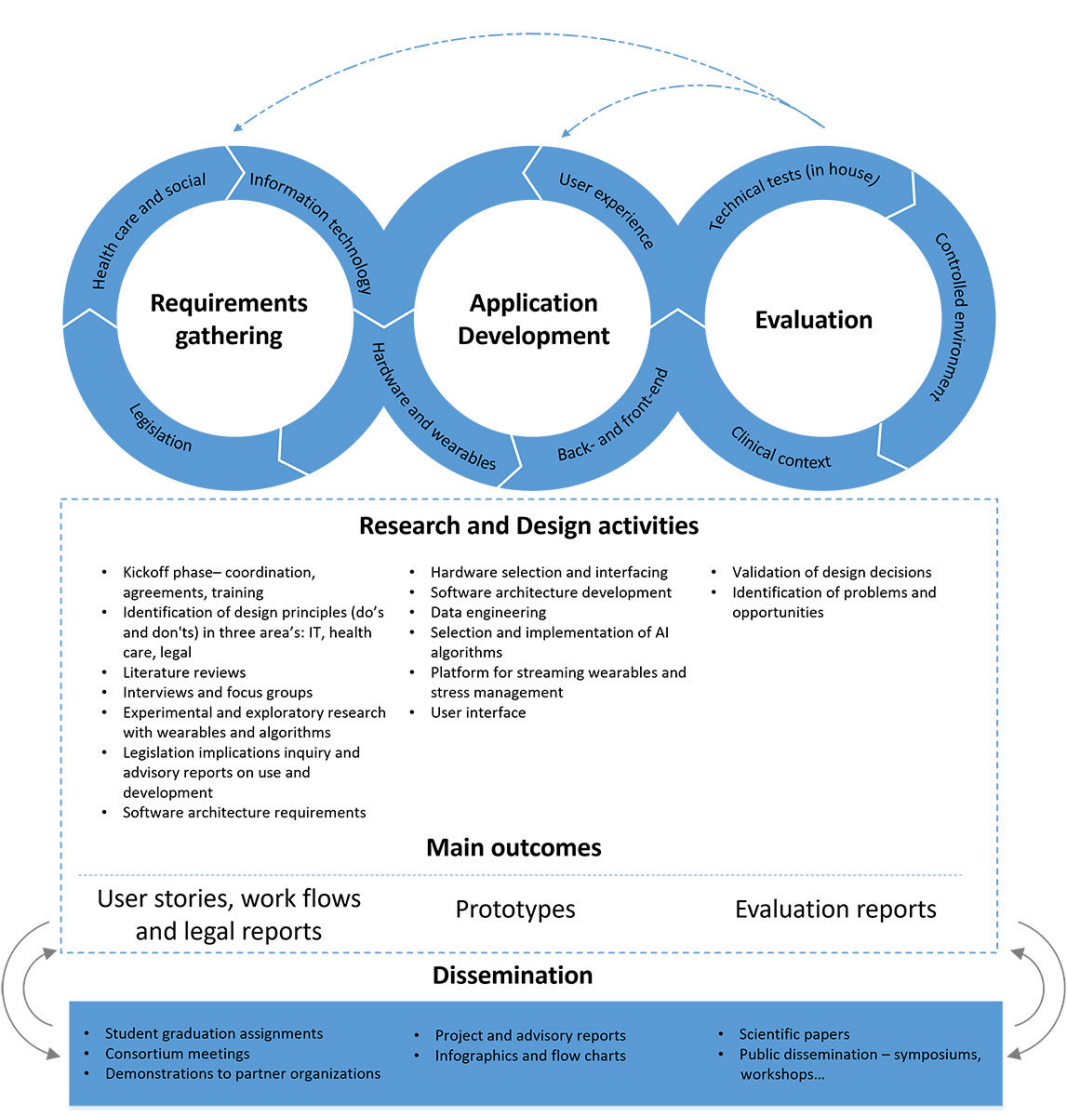
Research framework of the Sensors2Care project, including research and design activities. The project followed an iterative, transdisciplinary process structured around three key components: (1) requirements gathering, (2) application development, and (3) evaluation. Each component integrated expertise from health care, information technology, and law to address technical, clinical, and regulatory aspects. Activities included stakeholder consultations, exploratory wearable studies, legal analyses, and software development. Project outcomes (user stories, prototypes, and evaluation reports) were continuously refined and validated. This framework provided a structured approach to developing stress-monitoring wearables for vulnerable populations.

**Figure 2.**
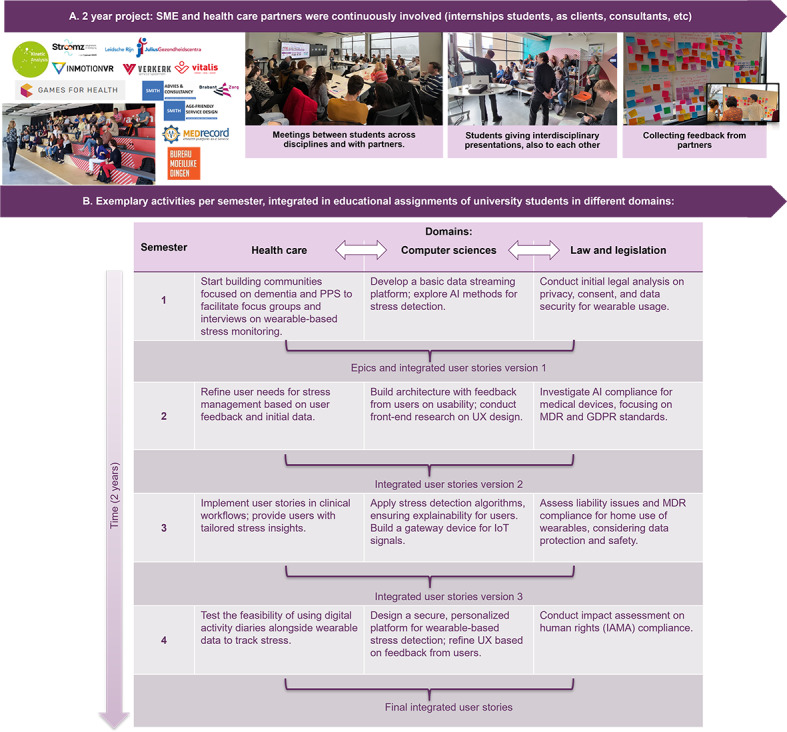
Operationalization of TDA within a learning community involving students, university researchers, health care professionals, and industry partners. (**A**) Stakeholders (small and medium enterprises and health care partners) were continuously engaged, together with students and university researchers. (**B**) The 2-year project was aligned with academic semesters to maximize student participation. In semester 2, epics and initial integrated user stories were defined collaboratively. At the end of each subsequent semester, interdisciplinary results and deliverables were synthesized into updated user stories, informing the subsequent semester. Through 4 iterative cycles, this process ensured continuous interdisciplinary refinement, leading to validated user stories and prototypes. AI: artificial intelligence; GDPR: General Data Protection Regulation; IoT: Internet of Things; MDR: Medical Device Regulation; PPS: persistent physical symptoms; SME: small to medium enterprise.

The research activities followed a structured, iterative process designed to facilitate co-learning and knowledge integration among health care, computer sciences, and law [34]. All phases, from requirements gathering to application development and evaluation, were informed by diverse expertise and stakeholder input. The overall research framework is illustrated in Figure 1 and elaborated in “research activities.” Researchers provided continuity and methodological guidance, while students participated as active contributors through graduation projects and internships. Their participation was supported by the university’s programmatic assessment framework [36,37], an educational approach that emphasizes continuous feedback and reflection rather than single-point evaluation. Within this system, students collected feedback from multiple stakeholders in the LC, including professionals, researchers, and end users, thereby fostering reflexivity and knowledge exchange in line with TDA’s principles [27,28,33]. The LC structure (Figure 2) combined recurring student participation with stable involvement of researchers and non-academic partners, ensuring effective knowledge transfer and continuity over time. This rhythm supported iterative cycles of learning and reflection. Each semester, 3 LC-wide meetings facilitated cross-sectoral collaboration and iterative feedback: at the outset (orientation and expectation management), midway (joint evaluation and refinement), and at the conclusion (integration and dissemination of outcomes). Additionally, smaller groups within the LC met in various configurations as needed over time to address specific research activities. Together, these structured and flexible interactions maintained coherence and fostered ongoing joint reflection throughout the process, consistent with reflexivity as a continuous, collaborative practice within transdisciplinary research [35].

### User Stories as a Methodological Instrument

As this project aimed to develop prototypes that are informed by clinical, technical, and legal perspectives, a structured approach was required to support transdisciplinary collaboration and integrate diverse expertise. Therefore, we adopted a method inspired by agile software design practices [38], enabling the translation of broad project goals into actionable design and research activities.

“Epics” were introduced as overarching themes capturing the main development goals. Two epics were established: one for individuals with dementia and their caregivers, and another for patients with PPS and their physical therapists. These epics were defined during a dedicated stakeholder meeting involving academics, health care professionals, and representatives from the involved SME. Broad themes were collaboratively formulated based on stakeholder expertise and clinical experience. Subsequently, each epic was broken down into user stories.

A user story, traditionally used in software development to document system functionalities, describes a concrete need or requirement from the perspective of a particular user, following a standardized format [38]: as <user role>, I want <functionality> so that I <goal>. For example, “As an individual with PPS, I want to access a weekly overview of stress moments, so that I understand and can discuss my stress levels with my physiotherapist.”

In this project, user stories were adapted as a transdisciplinary tool for reflection, integration, and iteration within the LC. Their structured format enabled diverse stakeholders, including health care professionals, patients, students, IT developers, and legal experts, to refine requirements, align technical and ethical considerations, and ensure the practical relevance of the prototypes.

By synthesizing insights from various research activities (Figure 1), including literature reviews, focus groups, interviews, legal analyses, and prototype evaluations, user stories provided a structured yet flexible method for integrating stakeholder needs throughout the project (Figure 2). The thematic clustering and final synthesis of user stories, including their role in guiding prototype development, is discussed in “Analysis: Synthesizing user stories.”

### Research Activities

Within the TDA context of this project, mixed methods were applied [39], and findings were triangulated to integrate insights from different disciplines into the development and refinement of user stories regarding wearable-based stress-monitoring for individuals with dementia and PPS. General principles of participatory design research [30,40,41] were applied in an iterative process, primarily covering co-explorative and conceptual design stages. Research and design activities took place in three defined main components: (1) requirements gathering, (2) application development, and (3) evaluation (Figure 1). These components ran in parallel and informed one another, although the primary focus shifted over time. In the initial phase, the focus was on initial requirements gathering (from a health care, social, legal, and technical perspective), with limited prototyping and evaluation. As time progressed, the focus shifted toward prototyping and evaluation, while requirements activities were mainly aimed at refinement.

Expertise feedback and requirements from 6 SME’s (ie, IT companies developing software) and 4 health care institutes were frequently (3 times per semester) collected during the LC meetings. During those meetings, students of different domains (health, computer science, and law), supervised by university researchers, presented their research and outcomes. Extracted user stories, developed prototypes, and evaluations were discussed in this transdisciplinary setting. Feedback collected during those meetings was applied to subsequent research activities. Next to sharing expertise and reflecting with all stakeholders together, these meetings were also intended to contribute to the feeling of cohesion.

As detailed in the following sections, research activities in this phase included literature reviews, focus groups, and interviews with people with dementia, PPS, health care providers, and caregivers, producing legal advisory reports, and starting with initial prototyping of the system’s infrastructure.

### Requirements Gathering

#### Overview

This first component focused on identifying user needs, legal considerations, and technical constraints related to stress monitoring using wearables. To this end, several scoping reviews, stakeholder interviews, focus groups, and legal research were conducted.

#### Interviews and Focus Groups

**Figure 3.**
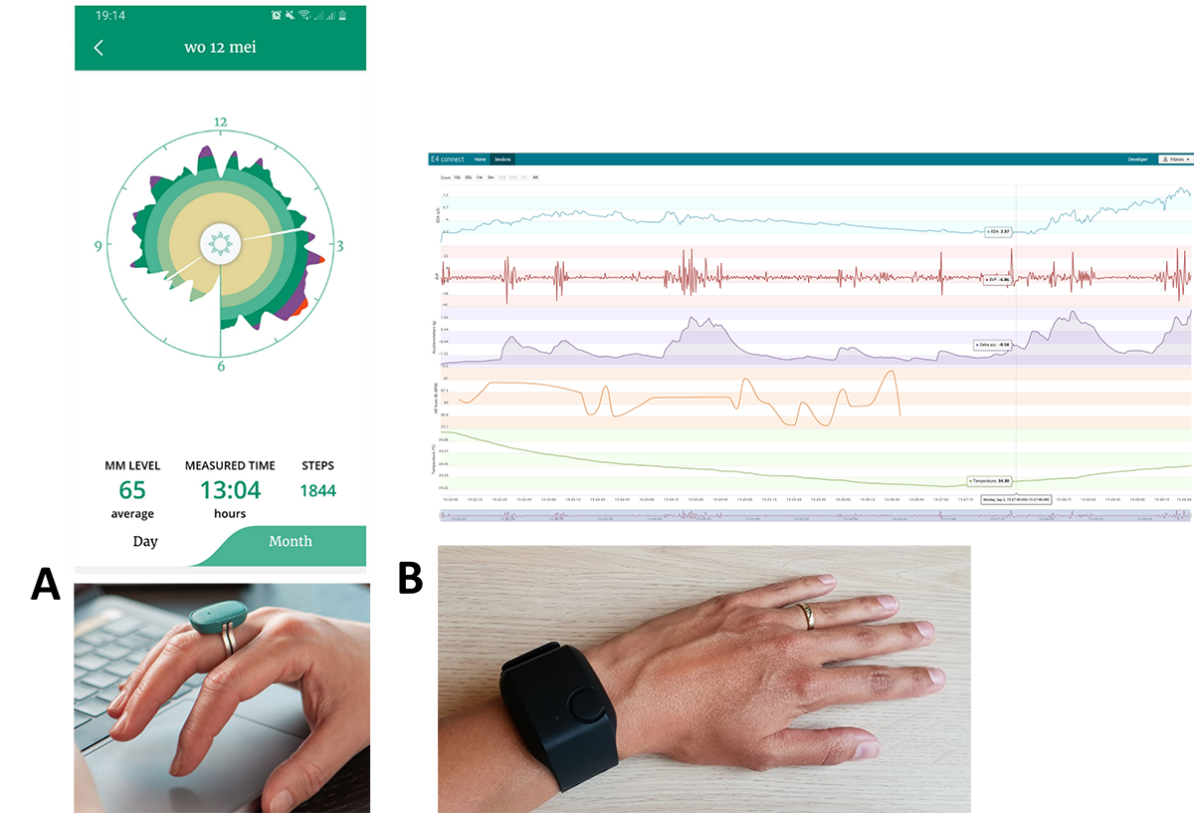
Off-the-shelf technology, which measures stress, was used as a probe to gather the first requirements of end users. (**A**) The Moodmetric ring with an exemplary visualization and (B) the Empatica E4 with an exemplary visualization.

Throughout the project, focus groups and individual interviews were conducted with stakeholders. For the dementia case, 14 sessions were conducted with health care professionals, 5 with informal caregivers, and 10 with people with dementia. For the PPS case, 11 sessions were held with physiotherapists and 11 with patients. During the first interviews and focus groups, prototypes were not available yet. Instead, existing visualizations from off-the-shelf technology (Figure 3) were used as probes during the interviews and focus groups, such as the Empatica E4 (Empatica Inc., Milan, Italy) and the Moodmetric ring (Vigofere Ltd., Helsinki, Finland). These probes were not presented as candidate solutions, but as a tool for participants to reflect on feasible possibilities in stress monitoring and articulate preferences in a more concrete way than abstract questioning would allow. Requirements emerging from those interviews and focus groups informed subsequent prototype development. Once prototypes were available, these visualizations were used as probes instead of off-the-shelf technology.

The complete methodology and outcomes of these interviews and focus groups are extensively detailed in a forthcoming research paper (van Westrienen et al, unpublished data, 2026). In the dementia use case, 2 focus groups were organized, including 5 health care professionals and 2 informal caregivers. Additionally, individual interviews were conducted with 3 informal caregivers, 6 health care professionals, and one person with dementia. For the PPS use case, individual interviews were held with 11 health care professionals and 13 PPS patients.

All interviews and focus groups followed a semistructured protocol [42], enabling both structured comparability and open-ended exploration. Discussions focused on perceived challenges and opportunities of wearable stress monitoring, ethical concerns regarding privacy and autonomy, and the feasibility of integrating such technology into existing health care workflows. To enhance reliability, member checks were performed, allowing participants to verify the accuracy of interpretations [43].

In addition to health care stakeholders, SME representatives from IT companies were actively involved in focus groups and interviews. Their participation ensured that technological feasibility, industry standards, and potential implementation challenges were taken into account from the early stages of the project. Discussions addressed data processing, sensor integration, and stress detection models; aligning technical capabilities with end user needs.

#### Legislation

Legal researchers were involved from the beginning of the project to provide early-stage advice, ensuring that legal considerations were integrated throughout the development process, rather than at the end when the prototype was (almost) finished. Key regulatory frameworks, including the General Data Protection Regulation, the Medical Device Regulation (MDR), the EU Artificial Intelligence (AI) Act, the Dutch Care and Compulsion Act, and Dutch intellectual property laws, were reviewed to ensure compliance from the outset. Legal advisory reports were generated, outlining potential constraints and ethical considerations, which were then integrated into user stories and early system requirements. Additionally, a Fundamental Rights and Algorithm Impact Assessment was performed to evaluate broader societal implications. This assessment ensured that the solutions developed were not only legally compliant but also ethically sound and respectful of fundamental human rights.

#### Scoping Reviews

To complement empirical insights, 2 scoping reviews of the literature were conducted. The first review focused on AI-powered wearables for mental health, examining studies retrieved from PubMed, CINAHL, PsycNet, ACM Library, and ScienceDirect. The second review explored biofeedback interventions for stress monitoring in PPS patients, using PubMed, CINAHL, and PsycInfo. These scoping reviews were conducted by students under the researcher’s supervision. Their purpose was preparatory: to inform user-story development in this project rather than to serve as stand-alone systematic review outputs. Accordingly, the Preferred Reporting Items for Systematic reviews and Meta-Analyses extension for Scoping Reviews methodology [44,45] was applied as a guiding framework, but not all checklist items could be addressed in full (Checklist 1). Specifically, the reviews helped identify intersections between health care, social, technical, and legal requirements. User requirements that impacted the user stories of this project were extracted from the reviews.

### Application Development

**Figure 4.**
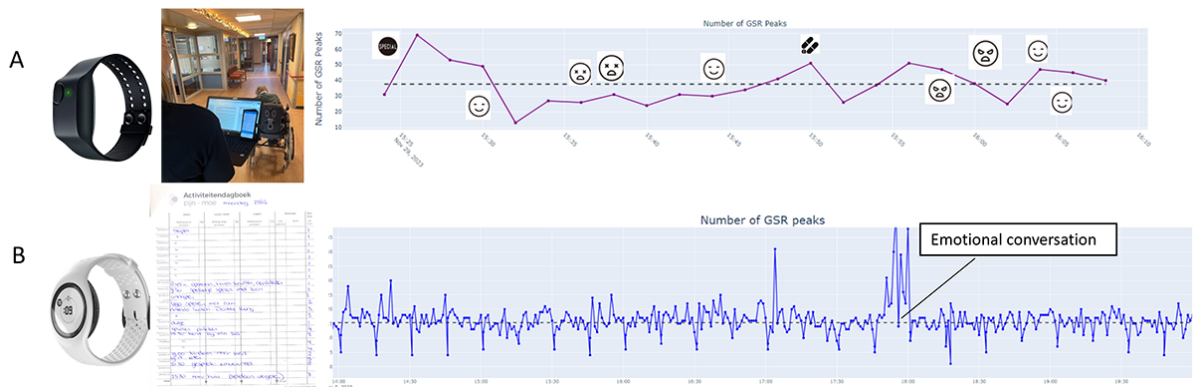
During field testing, different prototypes were tested. (**A**) A short (30 min) measurement on a person with dementia, combined with behavioral observations. By clicking on a pictogram, the user was linked to the observation notes of that time point. (**B**) A fragment (6 h) of a long-term 7-day measurement on a client with persistent physical symptoms, where a diary was kept as part of the usual care. In both prototypes, a semiautomated pipeline was used, in which data from the wearable was processed through a validated Trough-to-Peak (TTP) algorithm [50] and manually combined with behavioral observations or the diary.

This second component focused on translating user stories, informed by requirements gathering, into functional prototypes that were used for evaluation and subsequent refinement of user stories. Software prototypes of the back-end and front-end components were iteratively developed. The back-end architecture facilitates real-time data streaming, processing, and stress detection, integrating algorithms and cloud services. Details can be found in a dedicated paper [46]. Stress detection algorithms were developed using the open-source and validated WESAD dataset [47], applying established feature extraction of physiological parameters, including skin conductance components [48]. Regarding the front-end, physiological data were visualized in line diagrams using the Plotly library (Figure 4 [49]).

As hardware development was beyond the project’s scope, we integrated existing commercial wearables, the Empatica E4 and EmbracePlus devices, into the prototyped platform. Both devices, which are medical-grade wearables with CE marking and designed for wrist wear, offer comprehensive access to raw signal data. They measure peripheral skin temperature, electrodermal activity at 4 Hz, movement via a 3-axis accelerometer (ranging from 32 Hz in the E4 to 64 Hz in the EmbracePlus), and heart rate and blood pulse volume through photoplethysmography with 1-channel (E4) or 4-channel (EmbracePlus) at 64 Hz.

During prototype development, the Technology Impact Cycle Tool (TICT; [51]) was used as part of the computer science curriculum. TICT supports designers to estimate and understand the impact of new technologies, and critically think about privacy, data, human values, and sustainability.

### Evaluation of Prototypes

Developed prototypes (Figure 3) were evaluated using interviews after real-life experiences with those prototypes. For dementia, the Empatica E4 wristband was used (Figure 4A). Ten people with dementia living in a nursing home were selected by the nursing staff as residents with challenging behaviors, eligible for wearing the sensor. Depending on the subject, the sensor was worn for 30 to 60 minutes. After processing the data, caregivers were presented with stress measurements (using the developed prototype) combined with behavioral data observed by the researchers and interviewed to gather their insights and opinions about their experience with the stress measurements.

In the case of PPS, the EmbracePlus (Empatica Inc) wristband was used (Figure 4B). Twelve individuals with PPS who were treated by a physiotherapist wore the wristband continuously for a week. While they had the option to wear it 24/7, participants were free to remove it for charging or in situations where they felt uncomfortable being measured. Additionally, participants kept a paper diary as part of the usual care. After processing the data, individuals with PPS and their physiotherapists were presented with stress measurements per day (using the developed prototype; Figure 4B) and interviewed to gather their insights and opinions about their experience with the stress measurements.

### Analysis: Synthesizing User Stories

After it was established that extracting new user stories (eg, during focus groups and interviews after field testing, see Figure 2) did not yield new information (ie, saturation occurred), a dedicated working session was organized to categorize the user stories (197 in total) into themes. As the project was situated in the co-exploration and conceptual design stages of an iterative, participatory process, the analysis approach, in line with TDA principles, emphasized joint reflection and integration of perspectives [28] rather than the resolution of differences [34].

To organize the large number of user stories collected from various sources, we grouped them thematically according to user context. This clustering helped to identify shared needs, preferences, and challenges, supporting user-centric design, prioritization, and decision-making throughout the development process. To this end, at the start of the working session, separate lists were compiled for the PPS (136 user stories) and dementia (61 user stories) contexts. For each user context, 2 dedicated analysis groups were formed (making 4 groups in total). Each group consisted of at least 3 LC members, including both health care and computer science experts involved in the project. These groups independently categorized the user stories within their respective context, creating thematic clusters. Subsequently, the themes were compared within and across groups (peer review), leading to a shared understanding and final consensus on the central themes derived from the user stories. This stepwise process emphasized joint reflection and integration of perspectives rather than the resolution of all differences, ensuring that multiple stakeholder viewpoints remained represented in the final thematic structure. To enhance shared understanding and final consensus, these central themes were subsequently discussed in LC-wide meetings.

### Reflection in the Learning Community

Important elements of (our) transdisciplinary work were sharing information, iteration, and reflection. As such, we reflected with the students involved in the project, team members, and partners from various domains (ie, health care, law, and computer sciences). We obtained feedback on the following three questions: (1) Reflecting on the last 2 years, what do you see as the main successes or benefits of working in a transdisciplinary learning community? (2) What did you experience as the main challenges or difficulty? (3) Would you recommend working in such a transdisciplinary learning community in the future? If so, what would you improve or do differently? The written reflections were analyzed qualitatively by 2 researchers (IM and MPS). Responses were reviewed and thematically grouped according to the structure of the 3 reflection questions. Recurrent ideas and notable formulations were identified and summarized to capture shared experiences.

### Ethical Considerations

The local board of research and ethics (Fontys Commissie Ethiek van Onderzoek) reviewed the use of wearables in PPS “86peeters30082022,” and the Medical Ethical Board of Brabant reviewed the use of wearables in dementia “NW2021-45.” All procedures were conducted in accordance with institutional and national ethical standards and with the Declaration of Helsinki. Participants were adults; no financial compensation was provided. All identifying data were anonymized by the researchers and processed confidentially. All participants or their representatives were informed that participation was voluntary and that they could withdraw at any time without providing a reason. All participants in this study gave their informed consent after receiving verbal and written information.

In nursing homes, formal caregivers and legal representatives of residents with dementia provided proxy consent in accordance with institutional guidelines. Procedures included ongoing assessment of assent and comfort. A professional caregiver who knew each participant well assisted with putting on and taking off the wearable device and observed for any signs of distress, discomfort, or resistance. Any such behavior would have been interpreted as a withdrawal of consent. No indications of non-consent were observed during the study.

## Results

### Overview

We report the main findings of each research activity that impacted the user stories. Finally, we show an integration of the results in the formation of the user stories, and a reflection by the LC members regarding the TDA.

### Epics

The following epic was formulated for the dementia use case:

*The goal is to support the clinical reasoning of healthcare professionals to optimize the quality of life for a client (person with dementia and challenging behaviors). For this purpose, personalized insights are gained during stress moments by biometrics. The healthcare professional or caregiver uses a wearable as a supportive tool during a period in which the client is observed. The combination of the data can be analyzed by a behavioral expert (healthcare professional) afterwards to take a substantiated decision*.

The following epic was formulated for the PPS use case:

*The goal is to support the clinical reasoning of the healthcare professional in order to optimize the quality of life for the client (person with PPS). To this end, an adult with PPS (client) wears a wearable for up to one week during the activity registration in the treatment period with the physiotherapist (healthcare professional). This way, insight into the client’s stress moments is obtained through biometry, along with the activity registration. The physiotherapist can then discuss this stress measurement with the client afterwards, along with the activity registration, so that both gain insight into the relationship between exercises and activities with stress and relaxation and its impact on the experience of the symptoms. This insight can be used to make informed decisions during the treatment period for the therapist and support sustainable behavior change for the client*.

### Requirements Gathering

#### First Interviews and Focus Groups

During the first requirements gathered from end users, using off-the-shelf technology as a probe, the following needs were indicated. No differences were found between dementia and PPS. In both use cases, patients and informal caregivers indicated they want to obtain insight into stress levels, specifically in relation to an individually predetermined measurement goal. They also wanted the ability to share data with specified formal and informal caregivers. Furthermore, they emphasized the importance of being able to wear the device long-term during daily life and to integrate the technology into care as usual. Esthetic, non-stigmatizing wearables were preferred. In addition, users wanted the option to modify notifications and to add contextual information, such as perceived stress or relevant events.

#### Legislation

The legal advisory reports produced during the project provided detailed insights crucial for the development of user stories and prototypes. Specifically, the following points were addressed:

- MDR (EU): The advisory report identified that the wearables used most likely fall under risk class IIa, necessitating compliance with specific safety and performance requirements. This included conducting risk assessments, implementing a quality management system, and ensuring post-market surveillance. Additionally, informed consent procedures had to be meticulously followed, with clear communication to participants about the wearable’s purpose and data handling.
- General Data Protection Regulation (EU): The reports emphasized the need for robust data protection measures. This involved obtaining explicit consent for data processing, pseudonymizing personal data at the earliest opportunity, and maintaining a data processing register. Data portability and the right to withdraw consent were also highlighted as essential compliance elements.
- AI Act (EU): The advisory report identified that the AI algorithms used most likely fall under the category “high risk” (because of the use of AI algorithms within a medical device), necessitating compliance with specific safety and performance requirements. Compliance with the AI Act required ensuring that the AI algorithms used in stress detection were transparent, explainable, and non-discriminatory. The legal advisory highlighted the importance of conducting regular audits and impact assessments to monitor the ethical deployment of AI.
- Care and Compulsion Act (Dutch): The project had to navigate the legal requirements for using wearables in the context of dementia, especially regarding compulsory care. The advisory recommended implementing the use of a decision-making framework to ensure that any compulsory use of wearables was justified and legally compliant, and to involve legal representatives in the consent process where necessary.
- Dutch Intellectual Property Laws: The reports provided guidance on protecting the intellectual property generated during the project and on preventing infringement of third-party IP rights. This included ensuring that all contributions from various stakeholders were appropriately credited and protected, also when open source software is used for developing the application.

These specific legal insights were integral to the user stories, ensuring that each phase of prototype development was compliant with relevant regulations. For example, the user stories for health care professionals included requirements for transparent data handling and the ability to easily obtain and manage patient consent, while those for developers focused on embedding regulatory compliance into the design and functionality of the wearables.

#### Literature Reviews

Overall, the 2 reviews identified the prevalent use of wearable devices, particularly those worn on the wrist. However, significant gaps were found in the literature regarding acceptability, user experience, and usability, all of which are crucial components for the development of user stories. Many studies focused on the development and accuracy of machine learning algorithms, while aspects related to user acceptability, such as real-life experience, comfort, and ease of use, were addressed far less frequently. Regarding usability, participants expressed preferences for comfort and discretion in wearability, with some suggesting that integration into clothing or shoes would be beneficial. Personalization was also valued, with preferences for distinctive features such as colored or differently textured bands to identify one’s own device. Additionally, participants appreciated being able to track information such as their step count on the wearable device. Practical difficulties were reported, such as having to remove the device for charging or bathing, as well as annoyance caused by audible beeps emitted by the device. Moreover, the limited inclusion of long-term follow-up data in the studies restricts insight into sustained use and user experience over time.

### Evaluation of Developed Prototypes

The final prototypes used for evaluation in this project are shown in Figure 4. These prototypes were exploratory artifacts developed to translate user insights into tangible design directions rather than finalized products.

In the dementia case, formal caregivers considered the measurements useful. They indicated that the short-term graph with annotations (prototype Figure 4A) provided a clear overview. Caregivers appreciated the use of neutral blue colors and the inclusion of pictograms that served as hyperlinks to the observational log corresponding to the same time points. To be able to work effectively with the data, they explicitly expressed the need for additional information or training on how to interpret the measurements.

Patients with PPS (prototype Figure 4B) and their caregivers experienced the measurements as useful. They appreciated the neutral blue color of the graph, the daily overview of measurements, and the interactive nature of the Plotly graph (ie, the ability to zoom in and out on specific time periods during the day). As an improvement, patients and caregivers expressed a wish for an additional weekly overview in a single graph, as well as the ability to add contextual information digitally. Importantly, some patients indicated that they found it difficult to interpret and apply the measurements and explicitly expressed a need for more information or training. Most participants disliked the aesthetic appearance of the EmbracePlus device. Notably, after viewing the prototype, patients and caregivers expressed differing views regarding notifications: patients stated they would like to receive notifications about stress moments, either in real time or as a daily summary, whereas caregivers were more hesitant, noting that this could lead to an unhealthy focus on stress. Caregivers emphasized that they want to control the notification settings for their patients to ensure that measurements do not interfere with treatment.

### Final User Stories

#### Overview

Analysis of the final set of user stories, which were developed through multiple iterations (Figure 2) within the framework of the 2 epics, revealed the same 7 themes for both the dementia and PPS contexts. These themes portray the needs and requirements of the users. The order of appearance does not reflect the relative importance of the themes. For each theme, it is described how it was reflected in the conceptual prototypes that were evaluated as part of the co-exploration and conceptual design stages.

#### Data Insight and Analysis

This theme revolves around displaying the data collected by the wearable. Users seek a clear and straightforward overview of the data, enabling them to quickly grasp both short-term and long-term trends and draw conclusions efficiently.

*As a nurse, I want to see the data of multiple days, so that I can easily recognize patterns*

[Health care professional dementia]

*As a person with PPS, I want to use a wearable 24/7 so that every situation in my daily life can be taken into account*.

Both in the dementia and the PPS context, the importance of being aware that stress can also be something positive was acknowledged:

*As a person with PPS, I want the color to show my stress level not to be red because stress is not always negative.*

This theme was reflected in the conceptual prototypes by displaying daily and weekly overviews of stress levels, in a neutral color scheme (Figure 4), allowing users and their therapists to explore both short- and long-term trends.

#### User Input (Context)

In this theme, the focus is on adding context and feedback to the physiological data. Users want to express their experiences and feelings related to stress and relaxation, using notes, smileys, or scores.

*As a nurse, I want to be able to report what caused the peak and at what time it occurred when dealing with relaxation and stress.*

[Health care professional dementia]

*As a physiotherapist for a person with PPS, I want patients to be able to pick between five different smileys to measure their stress level so that I have more context about the stress level*.

In the conceptual prototypes, contextual input was collected using paper-based methods that reflected existing care practices of the participants (ie, care as usual). In the dementia case, caregivers used observation forms with time-stamped notes to record behavioral events, which were later linked to the physiological stress data in the prototype using clickable pictograms (Figure 4A). In the PPS case, participants kept a paper diary during field testing to annotate situations that caused or relieved stress (Figure 4B), which was later discussed alongside the physiological data.

#### Notifications

This theme addresses receiving notifications or alerts from the wearable or app when stress levels become too high or when action is needed. Users also desire the ability to customize notifications based on their preferences and needs (including sensitivity and specificity, to influence the likability of false positives and negatives).

Both health care providers for people with dementia, as persons with PPS, want a notification function to signal increased stress levels:

*As a nurse, I want the wearable to be able to prevent stress. It should provide a notification before stress reaches its peak, so that healthcare professionals can take action before the stress becomes too intense.*

[Health care professional dementia]

*As a person with PPS, I want to receive a signal from the device when the stress peak happens because then I can be ready or take some actions with it to prevent the complaints caused by the stress*.

However, health care professionals do not all agree with a notification feature for their patients:

*As a physiotherapist for a person with PPS, I want to have a turn-off notification from my patients so that I do not have to get every stress peak from my patients, and I can avoid an unhealthy focus on their stress*.

This theme was discussed but deliberately not implemented in the conceptual prototypes due to ethical and practical considerations. While some PPS clients expressed interest in receiving real-time stress alerts or daily summaries, others, especially therapists, preferred to avoid notifications to prevent fixation or anxiety. As a result, data were reviewed retrospectively together with an expert, enabling participants to reflect on stress episodes without triggering immediate reactions during daily life.

#### Data Access and Sharing

How does the data from the wearable/app travel to its final destination (eg, cloud storage), and who can access it? Users want control over who can view and use their data. In the PPS context, it was furthermore explicitly found that users also want to transfer data quickly, securely, and reliably from the wearable to the client's or health care professional's records or system. Additionally, people with PPS want to discuss the data with their physical therapist to improve and support their treatment.

*As a nurse, I want to share the results of the stress measurement with legal representatives of the person with dementia, so I can keep the family up to date.*

[Health care professional dementia]

*As a person with PPS, I want the healthcare professionals to have an authority to look into the data because then I can see if a treatment is helpful for me or not*.

Also, users were conscious about their privacy:

*As a person with PPS, I want to be asked for the permission for my treatment so that I can keep my privacy*.

This theme was reflected through the development of a decision-making matrix to clarify data access rights, especially in dementia care, where legal representatives are involved. Additionally, in dementia care, a tool was developed to ensure compliance with the Dutch Care and Compulsion Act when wearables were used.

#### Wearable (Hardware)

Users expect a device that is comfortable, durable, and easy to use.

*As a nurse I want a wearable which is made of soft material, so that (skin) irritations in people with dementia are prevented*.

[Health care professional dementia]

*As a person with PPS, I want the wearable to be durable and compact, so that I can use it in various treatments such as exercise therapy*.

This theme was reflected in the selection of the Empatica E4 and later the EmbracePlus devices, as these are comfortable, durable, and wrist-worn wearables that allowed practical evaluation of usability and long-term wear in both contexts.

#### Support

This theme focuses on providing information and support to users of the wearable and app. Health care professionals require clear instructions to ensure correct use of the wearable. Users want a manual or an FAQ page where they can find answers to their questions or issues. They also want the option to contact technical support if they need assistance.

*As a nurse, I want clear instructions, otherwise the wearable is perceived as too complex. The interest of healthcare professionals needs to be piqued first, after which a workshop can potentially be provided. This way, we know how to properly use the wearable*.

[Health care professional dementia]

*As a physiotherapist for a person with PPS, I want there be a short schooling for me and my colleagues about the use of the wearable so that I know how to use the wearable*.

This theme was reflected by providing short instruction sessions before data collection and on-demand support from a researcher during measurements, ensuring correct use and confidence among participants. This clear onboarding was essential; without it, caregivers hesitated to adopt the wearable due to perceived complexity.

#### Strategic Use of Wearable

This theme emphasizes purposeful use of the wearable. Users want the wearable to measure stress levels and assist in anticipating or managing stressful moments. This is crucial for preventive care as well as the adoption of the technology.

*As an informal caregiver I want to measure until we reached the goal of the measurement. It should be continuously evaluated if the measurement’s goal is reached and the person with dementia still needs the wearable*.

[Informal caregiver for someone with dementia]

*As a person with PPS I want an overview of my stress level during the day so it can help me recognize my stressful moments*.

This theme was reflected in the decision to use the wearables only in cases where stress monitoring could address a concrete care question, ensuring goal-oriented and meaningful application. In the PPS context, wearables were deployed episodically to help clients and therapists assess progress and behavioral triggers. In the dementia setting, the wearable was reserved for residents with challenging behaviors, allowing care teams to identify stress patterns and evaluate the timing or necessity of interventions.

### Reflection on the Learning Community and Transdisciplinary Approach

Several aspects were highly emphasized by many of the LC members. Dominantly, the possibility to learn from each other and learn how to better communicate with other disciplines was highly emphasized. LC members appreciated the willingness of others to be “open-minded,” “step outside their own knowledge bubble,” and to receive feedback and opinions from very different points of view. Several members further elaborated that the TDA created an atmosphere in which all parties are more engaged, motivated by the mutual work, which also pushes the project forward. Growth, in particular, of the students was also mentioned as a benefit of the mutual learning experience. Particularly, specific disciplines (eg, the legal perspective) can exchange and provide valuable input throughout all phases of the research and design process, rather than being involved only in later or specific phases. This created a sense of progression, and more importantly, as emphasized by many participants, allows a combination of knowledge and expertise to tackle larger societal challenges.

Nevertheless, TDA can also create challenges. The main challenge presented is related to the management of a large group, with many partners and phases. This also requires good coordination, time management, planning, as well as more meso-organizational flexibility and understanding from the University of Applied Sciences for these efforts. More so, specifically in our TDA project, new students were introduced each semester. While several students stayed involved in the project even after their graduation (as well as co-authors of this paper), the constant change of students required them to start over, re-educate themselves on how to communicate transdisciplinary, and created a challenge to transfer the accumulated knowledge in an organized way.

Notably, most LC members indicated that they are content with the conducted TDA. Many emphasized that, in their opinion, this should be the way to conduct research, especially when dealing with complex societal and applied issues. Participants expressed a high willingness to join new TDA projects in the future.

## Discussion

### Principal Findings

This Sensors2care project demonstrates the practical application of the TDA within LCs, responding to earlier calls to move beyond conceptual discussions of transdisciplinary research [32]. It specifically addresses the co-exploration and conceptual stages of participatory design, where reflection and alignment, rather than finalized technical solutions, are central outcomes. From the outset, sustained, iterative collaboration between academic and non-academic stakeholders, including health care, IT, and legal experts, enabled practical knowledge integration, collaborative reflection, and transparent synthesis throughout the project. Shared communication across stakeholders was operationalized through user stories, which supported alignment throughout the process. Generative probes and early prototypes further helped elicit feedback, guiding the development of proof-of-concept prototypes with practical implementation in mind.

By integrating perspectives from students, researchers, health care professionals, informal caregivers, and industry partners, the LC facilitated knowledge exchange and mutual understanding across disciplinary and sectoral boundaries. This setting enabled the co-development of a wearable-based stress-monitoring system tailored to the needs of vulnerable populations. The iterative, mixed methods approach helped refine specific requirements throughout the project phases, which included literature reviews, focus groups, field testing in which prototypes served as tangible prompts for stakeholder reflection and feedback [30], and thematic analysis of user stories. By structuring needs in an accessible, user-centered format, user stories enabled reflection and alignment across technical, clinical, legal, and experiential perspectives. Reflexivity, a core TDA principle described as awareness of the broader context and ensuring the coherence and compatibility between project components [28], was embedded throughout the process to align stakeholder input, ethical considerations, and evolving technical design.

This inclusive approach grounded technological design choices in the clinical, social, legal, and ethical realities of individuals with dementia and PPS, populations often underrepresented in wearable technology research. Although user stories of both populations showed shared requirements in wearability, data confidentiality, and ease of use, unique cognitive and physical challenges of each group necessitate customization within the design for the specific population. Themes like strategic use, notifications, user input, data insight, access, and sharing highlighted the importance of personalization aligned with the intended goals of stress monitoring. This finding aligns with previous research on the balance between user burden and customization [52]. The wearable (hardware) theme further confirmed the need for comfort and durability, consistent with prior research [53,54]. Importantly, these shared themes emerged during the early, exploratory stages of co-exploration and conceptual design. It is expected that these broad, high-level requirements will evolve further into context-specific differentiations during later development and implementation stages.

Together, these insights demonstrate how the transdisciplinary process can translate diverse perspectives into concrete design requirements. While results are specific to the dementia and PPS, the described TDA process suggests methodological transferability that could inform similar practice-oriented innovation projects, particularly in chronic health care domains where technological, clinical, and legal perspectives intersect. As such, this exploration and conceptualization phase illustrates how TDA can be adapted and applied in diverse health care innovation settings.

### Methodological Limitations

Several methodological limitations should be considered when interpreting the findings. Because the project focused on the exploration and conceptualization phases of the design process, evaluation activities were short and small in scale. This reflects the agile, iterative design process, which relies on rapid prototyping and brief evaluation cycles to generate timely feedback and inform subsequent iterations. Consequently, findings from this stage should be interpreted as exploratory rather than generalizable.

While participatory design principles were applied throughout the project, user engagement with prototypes was limited to relatively short periods (ranging from a single session to several weeks). This limits the assessment of long-term usability and acceptability. Users often find it difficult to anticipate how emerging technologies will affect their daily lives, and short-term use may not reliably predict future use [55]. Additionally, the frequency and impact of discrepancies between subjective experience and physiological stress measurements require further investigation. Overall, sustained longitudinal user engagement, especially when designing for vulnerable populations, should be a key focus of future research.

In addition, the use of off-the-shelf wearables in the early exploration phase may have influenced participants’ perceptions of technological possibilities. These devices were intentionally presented as generative probes to stimulate discussion and reflection, not as candidate solutions, and were later replaced by project-specific visualizations once available. To mitigate potential bias, multiple commercial devices were used and explicitly framed as illustrative examples rather than evaluation objects. Nevertheless, early exposure may have shaped participants’ expectations regarding functionality or design.

### Challenges and Reflections on Stakeholder Involvement

TDA’s principles [28], including knowledge unity, real-world problem orientation, and reflexivity, were central to our LC. Active participation of non-academic stakeholders, such as care providers, industry representatives, and legal experts, helped ensure that prototypes are adaptable to clinical practice and integrate ethical and legal considerations. For example, legal assessments informed data security and patient consent processes, which are pivotal when designing for vulnerable populations. The TICT [51] was used to assess the societal implications of wearable deployment, showing the value of tools that promote ethical and practical considerations during the design process. It is important to recognize that the MDR and the AI Act are relatively recent European Union frameworks. As such, they do not always provide concrete or prescriptive standards. Some legal interpretations in this project were necessarily anticipatory, based on current guidance rather than judicial precedent. In the coming years, further clarification is expected as case law and additional regulatory commentary emerge. This highlights the importance of continuous legal reflection within long-term innovation trajectories.

Despite the successful use of TDA principles within the LC, some limitations emerged. Key societal actors such as policymakers and insurance companies were not involved, constraining the exploration of systemic factors that influence adoption and scalability in real-life settings. Moreover, although early prototypes were tested in practice, the project did not extend to the evaluation of market-ready solutions or their long-term integration into health care systems. These limitations align with recognized challenges in the NASSS framework, particularly within the adopter system, value proposition, and wider institutional and societal context [26,28]. This highlights the importance of extending the LC model in future work to include a broader range of societal stakeholders to support sustainable implementation.

### Implications for Transdisciplinary Education

By involving students from health, legal, and computer sciences, a meaningful contribution to transdisciplinary research was provided. While student participation requires careful guidance to maintain research quality, our experience aligns with previous work showing that well-structured, real-life research projects can provide valuable learning experiences and produce outcomes of academic relevance when appropriate supervision is in place [56]. This project shows that TDA principles, such as the integration of diverse forms of knowledge, reflexivity, and addressing real-world problems [27,35], can be taught early in professional development. This observation aligns with recent work of Amelink et al [57], which conceptualizes how transdisciplinary learning environments foster complex thinking by engaging students from different disciplinary backgrounds in real-world, problem-based collaboration. Their findings reinforce our conclusion that the TDA helps students connect disciplinary perspectives, enhance creativity, and approach health care challenges more holistically. Embedding TDA thinking within curricula across disciplines prepares future professionals to tackle complex societal challenges collaboratively, beyond the scope of their own fields.

These observations highlight the need for further research into how educational institutions can structurally support the long-term integration of TDA principles beyond individual projects and sustainably embed them within curricula and institutional frameworks. Moreover, given the evidence that integrating technology into the education of health students facilitates technology adoption in clinical practice [58-60], future studies should explore how to optimize this effect across all relevant domains. Such efforts can help prepare students as versatile, technology-savvy professionals capable of contributing to transdisciplinary projects and creating sustainable solutions in health care.

### Conclusions

This project demonstrates that applying a TDA within an LC enables the integration of legal, technical, and health perspectives from the earliest stages of innovation. The collaborative LC structure, organized through recurring meetings and stakeholder engagement, fostered mutual learning and reflexivity. Early involvement of legal expertise helped ensure that design choices were not hindered by regulatory constraints but instead guided by legal possibilities. Technically, the iterative development of prototypes, grounded in user stories, allowed for early validation and continuous refinement of requirements. Methodologically, user stories proved to be a valuable instrument, not only as a design outcome, but also as a structuring tool to align interdisciplinary contributions. Together, these insights show that LCs can serve as valuable environments for transdisciplinary research, where students, researchers, professionals, experiential experts, and industry partners co-create solutions to complex health care challenges. While the findings are context-specific and stem from an exploration and conceptualization phase, the applied TDA approach illustrates potential methodological relevance for other health care innovation contexts where transdisciplinary collaboration is essential.

## Supplementary material

10.2196/75836 Checklist 1 Preferred Reporting Items for Systematic reviews and Meta-Analyses extension for Scoping Reviews checklist.

## Abbreviations

AI: artificial intelligence
FCEO: Fontys Commissie Ethiek van Onderzoek
FRAIA: Fundamental Rights and Algorithm Impact Assessment
GAI: generative artificial intelligence
LC: learning community
MDR: Medical Device Regulation
NASSS: Non-adoption, Abandonment, Scale-up, Spread, Sustainability
PPS: persistent physical symptoms
SME: small to medium enterprise
TDA: transdisciplinary approach
TICT: Technology Impact Cycle Tool
